# The surface tension of surfactant-containing, finite volume droplets

**DOI:** 10.1073/pnas.1915660117

**Published:** 2020-04-01

**Authors:** Bryan R. Bzdek, Jonathan P. Reid, Jussi Malila, Nønne L. Prisle

**Affiliations:** ^a^School of Chemistry, University of Bristol, BS8 1TS Bristol, United Kingdom;; ^b^Nano and Molecular Systems Research Unit, University of Oulu, 90014 Oulu, Finland

**Keywords:** aerosol, cloud condensation nuclei, cloud droplet number concentration, surface tension, surfactant

## Abstract

Surface tension influences the fraction of atmospheric particles that become cloud droplets. Although surfactants are an important component of aerosol mass, the surface tension of activating aerosol particles is still unresolved, with most climate models assuming activating particles have a surface tension equal to that of water. By studying picoliter droplet coalescence, we demonstrate that surfactants can significantly reduce the surface tension of finite-sized droplets below the value for water, consistent with recent field measurements. Significantly, this surface tension reduction is droplet size-dependent and does not correspond exactly to the macroscopic solution value. A fully independent monolayer partitioning model confirms the observed finite-size-dependent surface tension arises from the high surface-to-volume ratio in finite-sized droplets and enables predictions of aerosol hygroscopic growth. This model, constrained by the laboratory measurements, is consistent with a reduction in critical supersaturation for activation, potentially substantially increasing cloud droplet number concentration and modifying radiative cooling relative to current estimates assuming a water surface tension. The results highlight the need for improved constraints on the identities, properties, and concentrations of atmospheric aerosol surfactants in multiple environments and are broadly applicable to any discipline where finite volume effects are operative, such as studies of the competition between reaction rates within the bulk and at the surface of confined volumes and explorations of the influence of surfactants on dried particle morphology from spray driers.

## Significance

Atmospheric aerosol particles cool Earth’s climate by serving as cloud droplet seeds. This cooling effect represents both the single most uncertain and the largest negative radiative forcing. Cloud droplet activation is strongly influenced by aerosol particle surface tension, which in climate models is assumed equivalent to that of pure water. We directly measure the surface tensions of surfactant-coated, high surface-to-volume ratio droplets, demonstrating that their surface tensions are significantly lower than pure water but do not match the surface tension of the solution from which they were produced and depend on finite droplet size. These results suggest surfactants could potentially significantly modify radiative forcing and highlight the need for a better understanding of atmospheric surfactant concentrations and properties.

Atmospheric aerosols impact climate directly by scattering solar radiation and indirectly by serving as cloud condensation nuclei (CCN), affecting cloud albedo and precipitation patterns. Despite representing the largest negative component to radiative forcing, the aerosol indirect effect due to aerosol‒cloud interactions (ACI) is the least understood and has the largest uncertainty (1). For liquid-phase clouds, a significant contributor to this uncertainty is the challenge of predicting accurately the number of cloud droplets formed in the presence of a given aerosol population, updraft, and total amount of water available (i.e., at a given critical supersaturation). The critical supersaturation at activation is quantified by the Köhler equation (2) and is strongly dependent on the surface tension of the activating particle at the critical point. A surface tension lower than that of pure water decreases the supersaturation barrier to cloud droplet activation. Most large-scale models implicitly assume a surface tension of pure water for activating particles (3), but validating this assumption is challenging. Including the effect of a reduced surface tension can change the indirect radiative effect (*IRE*) by up to 1.1 W·m^−2^, of the same order as the best estimate for ACI radiative forcing (1, 4, 5). A recent cloud parcel modeling study also demonstrated that surface active organics can affect cloud microphysics, optical properties, and radiative effects (6).

Aerosol chemical composition measurements challenge the assumption that the surface tension of activating particles is equal to that of water. Organic aerosols contain surface active molecules in sufficient abundance to reduce the surface tension of macroscopic solutions (4, 7, 8), and these abundances could, in principle, be sufficient to maintain a reduced surface tension throughout activation (7–11). Such a reduction could increase CCN concentrations by an order of magnitude (12). Moreover, organic films may significantly alter the growth pathway of activating particles (13–15), and dynamic surface effects may be important (16, 17).

The major challenge to resolve the impact of surfactants on aerosol particle surface tension is to accurately account for a surface-to-volume ratio orders of magnitude larger for a finite-sized droplet than for a macroscopic solution. A high surface-to-volume ratio increases the fraction of the total molecules partitioned to the surface, which lowers the bulk concentration and reduces the solute effect in the Köhler equation. Such partitioning may fully or partially counteract the surface tension lowering effect of surfactants and must be considered when predicting particle activation (18–22). Accounting for this partitioning is challenging because few approaches directly measure aerosol particle surface tension (23–27) and so far none have investigated surfactant partitioning in detail. Most approaches infer surface tension from hygroscopic growth or critical supersaturation measurements (28). Moreover, many studies report only macroscopic solution surface tension measurements and do not consider surface-bulk partitioning effects (4, 7, 8, 16, 29). Consequently, predictions of surfactant partitioning in aerosol are not accompanied by direct experimental comparisons for model validation. Accurate models describing particle surface composition are increasingly required (30), not only for predicting cloud droplet activation but also for understanding interfacial chemistry at the particle surface, which is emerging as key to a range of atmospheric processes (31–33).

We report direct surface tension measurements on picoliter droplets containing an atmospherically relevant primary solute and model surfactant, demonstrating that surfactant partitioning owing to the droplet’s finite volume is affected by droplet size and does not quantitatively match the macroscopic solution value. The experimental data validate a fully independent monolayer partitioning model for droplet surface composition, allowing a coarse estimation of the surfactant concentration range required to affect atmospheric cloud droplet activation. The results are broadly applicable to any discipline where the surface composition of finite-volume droplets is important, including resolving competition between surface and bulk reaction rates in confined volumes (34–37) and identifying surfactant effects on spray-dried particle morphology (38).

## Results

**Fig. 1.**
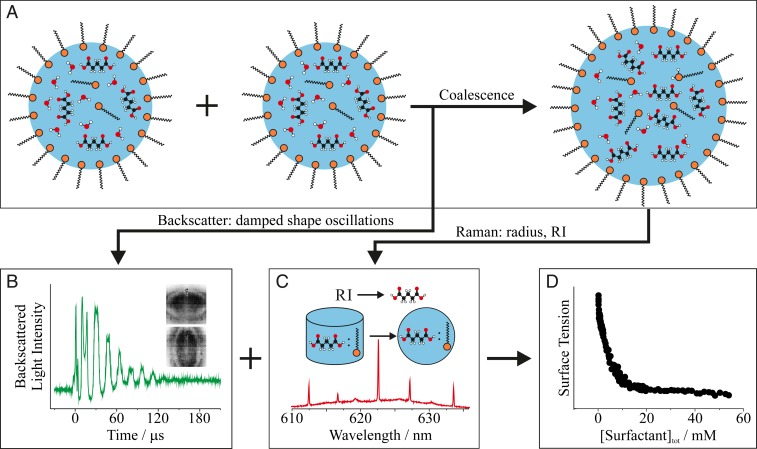
Conceptual description of the experimental procedure. (*A*) Two droplets containing a primary solute (0.9 M glutaric acid or 0.5 M NaCl) and the soluble surfactant Triton X-100 were optically trapped and coalesced. (*B*) The dynamic shape oscillations characteristic of the coalescence event were monitored by time-dependent changes to the backscattered light intensity. (*C*) The composite droplet size and refractive index (RI) were obtained by comparison of the whispering gallery modes in the Raman spectrum of the droplet to a library of Mie theory calculations. Parameterizations of RI and concentration give the primary solute concentration. The surfactant concentration is determined by assuming the primary solute:surfactant ratio in the nebulized solution is conserved in the droplet. (*D*) Together these data allow surfactant concentration-dependent measurements of the surface tensions of picoliter droplets.

Experiments were accomplished using a holographic optical tweezers instrument (23, 39, 40), and the experimental approach is conceptually illustrated in Fig. 1 (see *Methods* for more details). Two aqueous droplets each with a 5- to 10-μm radius containing known concentrations of a primary solute and a soluble surfactant were optically trapped and then coalesced into one composite droplet (Fig. 1*A*). Droplet coalescence excites damped oscillations in droplet shape (Fig. 1*B*), which were monitored by recording the elastic backscattered laser light. The frequency of these oscillations is related to the droplet surface tension (41, 42). Backscattered Raman light from the composite droplet (Fig. 1*C*) permits high accuracy and precision retrieval of the droplet radius and refractive index (RI). The RI is then used to determine the primary solute and surfactant concentrations.

The total droplet surfactant concentration [surfactant]_tot_ was quantified by assuming the relative ratio of primary solute to surfactant in the nebulized solution was conserved in the trapped droplets (see *Methods* for justification). The total droplet surfactant concentration is distributed between surfactant molecules at the droplet surface, *N*_surface_, and in the bulk, *N*_bulk_, and is described by

$${\left[surfactant\right]}_{\text{tot}}=\left(\frac{N_{\text{surface}}+N_{\text{bulk}}}{N_{A}}\right)\times \frac{1}{V},$$ [1]

where *N*_*A*_ is Avogadro’s number and *V* is the droplet volume. Therefore, the oscillation frequencies and the Raman spectra allow determination of the dependence of surface tension on surfactant concentration (Fig. 1*D*) from measurements on many aqueous solution aerosol droplets containing a fixed primary solute concentration (0.9 M for glutaric acid, a proxy for organic matter; 0.5 M for NaCl, a proxy for sea spray) and an incrementing surfactant concentration [Triton X-100, a well-characterized reference surfactant with properties comparable to those of some atmospheric surfactants (7, 20)].

### Surface Tensions of Droplets Containing an Organic Solute and Surfactant.

**Fig. 2.**
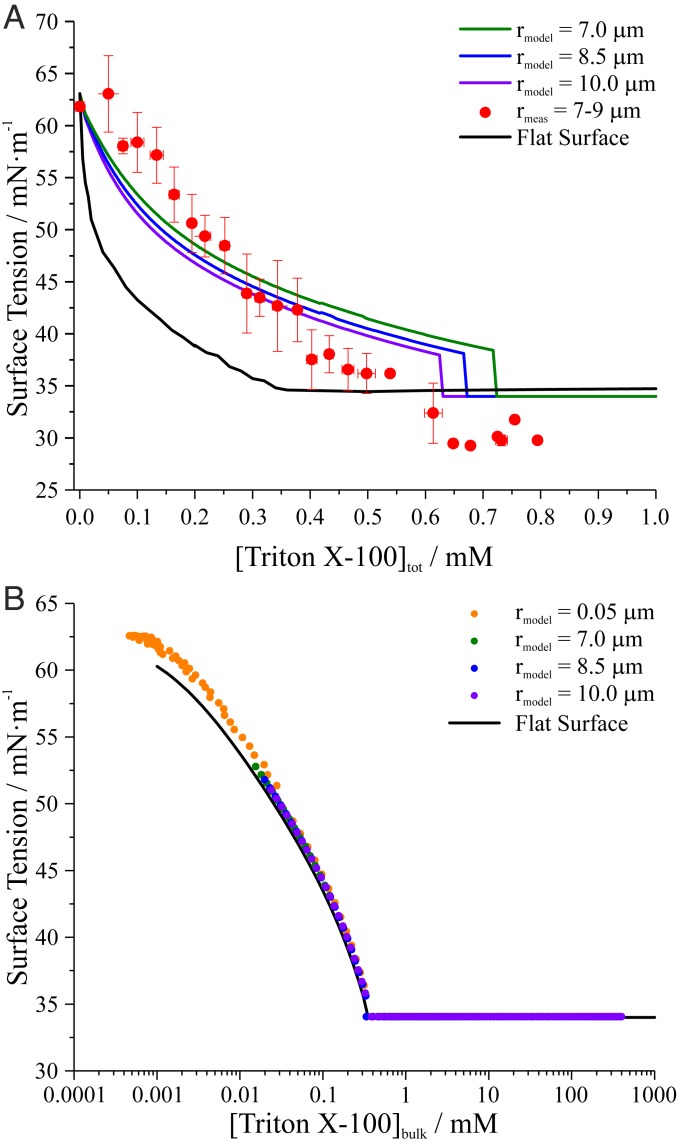
(*A*) Comparison of picoliter droplet (∼7- to 9-μm radius) surface tensions to macroscopic solution surface tensiometry measurements and monolayer partitioning model predictions at different droplet radii plotted as a function of [Triton X-100]_tot_ for droplets nebulized from an aqueous solution containing 0.9 M glutaric acid. Droplet measurements are averaged to 0.03 mM bins. Uncertainty bars represent the SD of the mean. (*B*) When monolayer partitioning model predictions are plotted as a function of [Triton X-100]_bulk_ (symbols), the results collapse onto macroscopic solution (flat surface) measurements (solid line), indicating that the model accurately considers surface-bulk partitioning in the droplets.

Fig. 2*A* presents the surfactant concentration dependence of surface tension for both picoliter droplet and macroscopic solution (flat surface) measurements for the glutaric acid–Triton X-100 system. Predictions of droplet surface tension using a monolayer partitioning model are also shown. The experimental droplet data are derived from 103 individual coalescence events and clearly show that measured values at a given [Triton X-100]_tot_ are up to ∼15 mN·m^−1^ higher than the corresponding macroscopic solution measurement. Consequently, the droplet measurements reach their minimum surface tension value at higher [Triton X-100]_tot_ than the macroscopic solution measurements.

The observation of higher measured surface tensions in microscopic droplets relative to the macroscopic solution is a consequence of the large surface-to-volume ratio of picoliter droplets. In a macroscopic solution, the fraction of surfactant molecules that partitions to the solution surface is negligible compared to the total number of molecules in the bulk because the surface-to-volume ratio is small and the total number of molecules in the solution volume is very large (i.e., *N*_surface_ << *N*_bulk_ in Eq. **1**, so *N*_tot_ ≈ *N*_bulk_, where *N*_tot_ = *N*_surface_ + *N*_bulk_). However, in a finite-sized droplet, the fraction of surfactant molecules that partitions to the surface represents a significant portion of the total number of molecules in the droplet because of the larger surface-to-volume ratio (*N*_tot_ >> *N*_bulk_) (19, 22). For example, a pendant drop tensiometry experiment on a 1 mM solution of surfactant might involve analysis of a 1-mm-radius droplet, which corresponds to a surface-to-volume ratio of 3,000 m^−1^. By comparison, a 10-μm-radius droplet has a surface-to-volume ratio 100 times larger (3 × 10^5^ m^−1^). Consequently, equilibrium partitioning of surfactant molecules to the surface of the 10-μm-radius droplet requires removal of a larger fraction of the total molecules from the bulk to form the surface film (i.e., a larger fraction at the surface), decreasing the fraction of molecules in the bulk and lowering [Triton X-100]_bulk_ below 1 mM. The result, when plotted as [Triton X-100]_tot_, is a higher surface tension in the droplet relative to macroscopic solution measurements. If surface-bulk partitioning were considered and an appropriate correction included, then the droplet measurements would collapse onto the macroscopic solution measurements when plotted as [Triton X-100]_bulk_.

The hypothesis that the droplet measurements are sensitive to the high surface-to-volume ratio and the resulting bulk depletion of the surfactant is tested using a monolayer partitioning model (43). This model was developed to quantify the surface-bulk partitioning for a droplet with a finite volume containing a specific number of surfactant molecules and is fully independent of the measurements (no fit parameters) (*Methods*). The surface is considered as a finite-sized phase with a thickness corresponding to one molecular layer, and surface tension is predicted from the bulk composition that results from the equilibrium distribution of molecules between the surface and bulk. Unlike other recently developed models, the only experimental data used to constrain the monolayer partitioning model are a modified Szyszkowski–Langmuir parameterization of the macroscopic solution surface tension data (*SI Appendix*, Fig. S1) and the solution density.

This monolayer partitioning model was selected over more commonly used Gibbs models (12, 22) and a two-dimensional (2D) van der Waals model (13) for several reasons. First, the monolayer partitioning model is fully predictive, has no free parameters, and is only constrained by measurements not related to the droplet experiments, in contrast to the 2D van der Waals model. Second, the Gibbs model tends to overestimate partitioning, as discussed in greater detail in previous modeling studies (17, 43, 44), and has compared poorly against experimental and field studies in some cases (12, 20), providing unphysically large estimations of surface layer thicknesses (20). Here, we compare the experimental data for the glutaric acid–Triton X-100 droplets to a Gibbs model (22) and, similar to these previous studies (12, 17, 20, 43, 44), we observe that the extent of surface partitioning is overpredicted relative to the measurements (i.e., the Gibbs model overestimates *N*_surface_ and underestimates *N*_bulk_), thereby predicting much higher surface tensions than experimentally measured (*SI Appendix*, Fig. S2). Although perhaps counterintuitive, this observation reflects the impact of surface partitioning on *N*_bulk_, which can usually be ignored in bulk phase measurements but strongly and implicitly depends on surface enrichment in finite-volume systems. The very act of enriching the surface depletes the bulk phase, which depresses the bulk surfactant concentration and leads to an erroneous inferred surface tension when the total composition alone is used as the reference concentration to predict surface tension.

Modeled droplet surface tensions are shown in Fig. 2*A* for three droplet radii (7.0, 8.5, and 10.0 μm), spanning the experimentally measured droplet radius range. The modeled surface tensions, which account for surface-bulk partitioning, agree well with direct droplet measurements. At [Triton X-100]_tot_ = 0 mM, the predicted droplet surface tension is equivalent to that of the macroscopic solution of glutaric acid. This observation indicates that depletion effects are not manifested in this size range for glutaric acid, which has weak intrinsic surface propensity but is a water-soluble organic compound, mainly due to the very high concentrations (hundreds of millimolar) studied. As [Triton X-100]_tot_ increases, the predicted surface tension decreases. Near [Triton X-100]_tot_ = 0.6 to 0.7 mM, the predicted surface tension sharply drops because the critical micelle concentration (CMC) is reached in the droplet bulk. This steep change in predicted surface tension is an artifact of plotting the model data against [Triton X-100]_tot_ rather than [Triton X-100]_bulk_. Beyond the steep change in surface tension, the CMC is surpassed and the surface tension is invariant with surfactant concentration.

The steep change in predicted surface tension results from the small size and small absolute number of molecules in a droplet, together with the manner of plotting the data. A small increase in the absolute number of surfactant molecules in the droplet bulk corresponds to a large increase in surfactant mole fraction, which can manifest as a steep change in surface tension for sufficiently small volumes. For example, the surface tension of an 11-nm-radius droplet is predicted to be initially unaffected by increases in [Triton X-100]_tot_ because all molecules are partitioned to the droplet surface and none reside in the droplet bulk. However, a single surfactant molecule residing in the droplet bulk drives [Triton X-100]_bulk_ above the CMC (43). Therefore, the predicted surface tension would implicitly follow a step function when plotted against [Triton X-100]_tot_, highlighting the importance of considering the total surfactant concentration rather than just the bulk concentration. However, as demonstrated in Fig. 2*B* and discussed below, when the experimental data are plotted against [Triton X-100]_bulk_, a continuous trend in surface tension is observed. For larger droplets, this behavior transitions toward a smoother variation in surface tension as the total surfactant concentration increases. The model prediction therefore qualitatively resembles the “compressed film-to-gaseous phase transition” of surfactant films described by a 2D van der Waals model (13, 14, 45). A key difference in our model is that the steep change in surface tension is due to size-dependent surface-bulk partitioning, whereas in the 2D van der Waals model it is due to a phase transition in the surface film (43).

To confirm the model accurately accounts for surface-bulk partitioning, surface tension predictions at 0.050-, 7.0-, 8.5-, and 10.0-μm radius were compared with macroscopic solution measurements (Fig. 2*B*). In this case, the model results are plotted as [Triton X-100]_bulk_ rather than [Triton X-100]_tot_ and follow the same trend, collapsing onto the macroscopic solution curve. This overlap indicates that surface-bulk partitioning is accurately accounted for by the model. (The ∼1 mN·m^−1^ discrepancy for the smallest droplets is due to surface-bulk partitioning of glutaric acid, not surfactant.) Moreover, a consistent smooth trend in surface tension occurs across all droplet sizes with increasing [Triton X-100]_bulk_, indicating that the steep change in surface tension observed in Fig. 2*A* is an artifact of representing the data in terms of [Triton X-100]_tot_ rather than [Triton X-100]_bulk_.

### Droplet Size-Dependent Surface Tension.

**Fig. 3.**
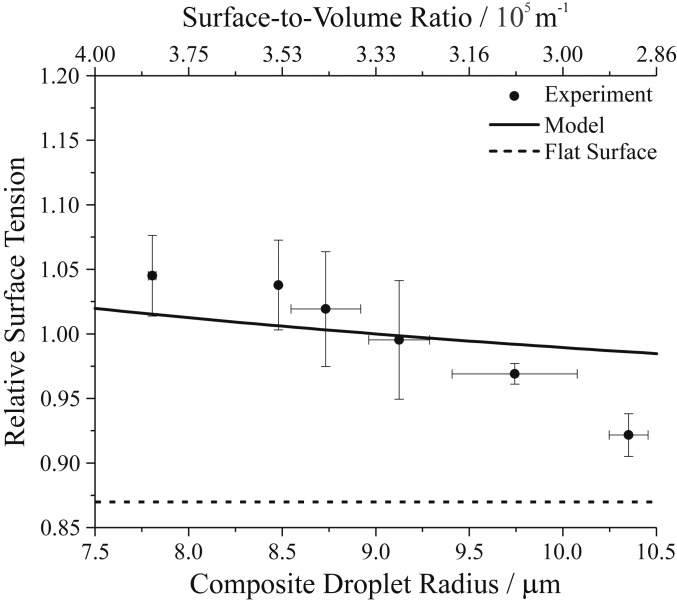
Size dependence of composite droplet surface tension for droplets produced by nebulization of an aqueous solution of 0.9 M glutaric acid and 0.42 mM Triton X-100. The solid line is the model prediction. A relative surface tension is plotted to superimpose the measured and modeled values. The relative value for γ_CMC_, the macroscopic solution value of surface tension at this concentration, is provided by the dotted line. The upper axis gives the surface-to-volume ratio for the droplet radii on the bottom axis. Data were averaged to 0.5-μm bins. Uncertainty bars represent the SD of the mean.

Fig. 3 reports surface tension as a function of droplet size for droplets nebulized from an aqueous solution containing 0.9 M glutaric acid and 0.42 mM Triton X-100, along with model predictions of the size-dependent surface tension shown as changes relative to the surface tension for a 9.0-μm-radius droplet. A trend of increasing surface tension with decreasing composite droplet radius and increasing surface-to-volume ratio is clear, confirming experimentally that the droplet surface tension is size-dependent at a constant [Triton X-100]_tot_. Although the same general trend is predicted by the model (higher surface tension at smaller radius), the magnitude of the change is smaller. Nonetheless, the hypothesis that surface-bulk partitioning is significant in picoliter droplets is confirmed.

### Surface Tensions of Droplets Containing Salt and Surfactant.

**Fig. 4.**
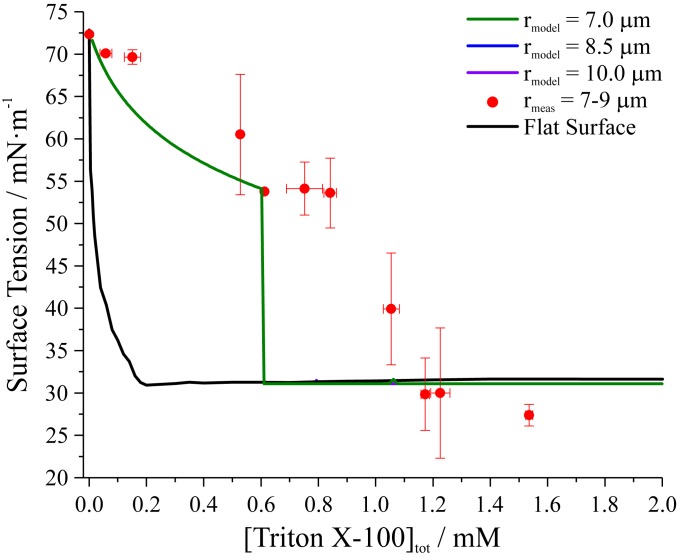
Comparison of picoliter droplet (∼7- to 9-μm radius) surface tensions to values from macroscopic solution surface tensiometry measurements and monolayer partitioning model predictions at different droplet radii plotted as a function of [Triton X-100]_tot_ for droplets nebulized from an aqueous solution containing 0.5 M NaCl. In practice, model results for different radii overlap each other. Droplet measurements are averaged to 0.1 mM bins. Uncertainty bars represent the SD of the mean.

Droplets containing NaCl and Triton X-100 were also studied. Fig. 4 compares surface tension measurements of 7- to 9-μm-radius droplets to macroscopic solution values. These data are derived from 39 individual coalescence events. The comparison of droplet and macroscopic solution surface tension values is broadly consistent with observations for the glutaric acid–Triton X-100 system: Picoliter droplet surface tension values are substantially higher than the corresponding macroscopic solution values when plotted against [Triton X-100]_tot_. A much larger maximum difference between droplet and macroscopic solution surface tension is observed for the NaCl–Triton X-100 system (35 mN·m^−1^) than for the glutaric acid–Triton X-100 system (15 mN·m^−1^). This observation is consistent with the salting-out effect. Salt alters surfactant partitioning properties, resulting in a larger surface excess (higher surface propensity) at a given [Triton X-100]_bulk_ for the NaCl–Triton X-100 system than the glutaric acid–Triton X-100 system (evidenced by the steeper slope of the surface tension vs. concentration curve for NaCl–Triton X-100 in *SI Appendix*, Fig. S3). Consequently, when droplet surface tension is plotted against [Triton X-100]_tot_, a larger [Triton X-100]_tot_ is required to reduce surface tension because a greater fraction of the surfactant molecules are partitioned to the droplet surface rather than the bulk. In other words, the relative magnitudes of *N*_surface_ and *N*_bulk_ in Eq. **1** shift toward *N*_surface_ for the droplets containing salt.

Model predictions of surface tensions for droplets (7.0-, 8.5-, and 10.0-μm radii) are also shown in Fig. 4. The surface tension values for each of these sizes are very close because of the stronger dependency of surface tension on [Triton X-100]_bulk_ compared to the glutaric acid–Triton X-100 system (*SI Appendix*, Fig. S1). Consequently, the dependency of droplet surface tension on [Triton X-100]_tot_ is weaker, decreasing the resolution of the partitioning model. The model captures the surface tension trend of picoliter droplets for the NaCl–Triton X-100 system qualitatively until the model predicts the CMC is reached in the droplet bulk around [Triton X-100]_tot_ = 0.6 mM with a steep change to a value of γ_CMC_. The disagreement between measurement and model is likely because partitioning is slightly underestimated by the model for this system.

To summarize, finite-sized picoliter droplets are shown to have surface tensions up to 35 mN·m^−1^ higher than the corresponding macroscopic solutions that produced them. Monolayer partitioning model predictions, which account for surface-bulk partitioning in finite-sized droplets and are fully independent of the droplet experiment, are in good agreement with the measurements. The good agreement between measurement and model suggests the general phenomena governing picoliter droplet surface tension are represented by the model.

## Discussion

**Fig. 5.**
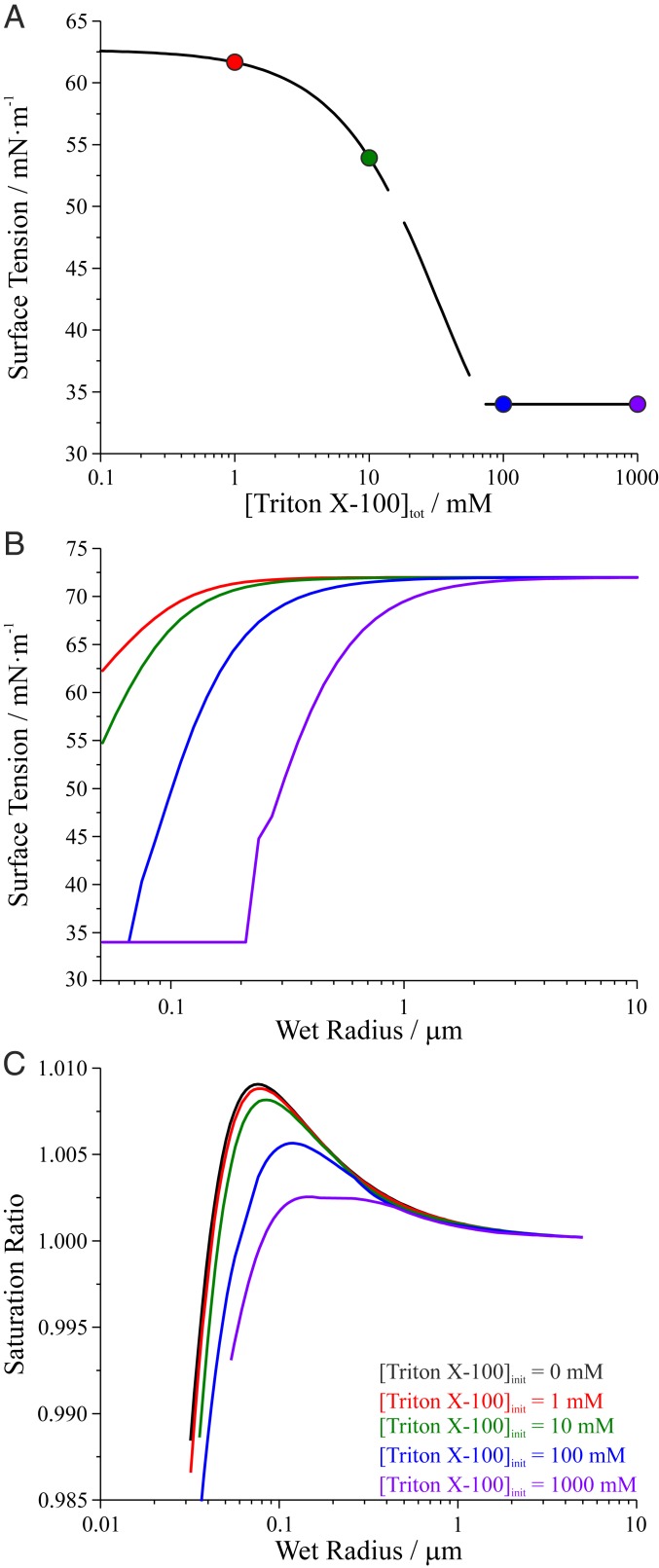
(*A*) Monolayer partitioning model predictions of the surface tension of a 0.05-μm-radius droplet as [Triton X-100]_tot_ is increased. (*B*) Surface tension predictions for droplets containing different initial [Triton X-100]_tot_ growing hygroscopically (i.e., by addition of water) from 0.05-μm to 10-μm radius. (*C*) Köhler curves describing cloud droplet activation for the same droplets. The different colored lines refer to droplets with initial compositions indicated by the colored circles in *A*. The predictions account for both dilution and the changing surface-to-volume ratio of the growing particle.

Partitioning models predict that accounting for the surface-bulk partitioning of surface-active molecules becomes increasingly important in smaller droplets, where surface-to-volume ratios are much larger than in micrometer-sized droplets (e.g., 6 × 10^7^ in a 0.05-μm-radius droplet). Based on this first direct validation of the model for picoliter droplets, we explored the surface tension effects of surfactants in smaller droplets more likely to dominate as CCN. Fig. 5*A* shows that for a 0.05-μm-radius droplet (a typical size for CCN) containing 0.9 M glutaric acid, [Triton X-100]_tot_ must be >>1 mM to substantially lower the surface tension, a consequence of a high surface-to-volume ratio.

Fig. 5*B* explores the significance of accounting for surface-bulk partitioning during cloud droplet activation by showing the hygroscopic growth of a 0.05-μm-radius particle with different initial surfactant concentrations. The model prediction accounts for both the dilution resulting from hygroscopic growth and the decreasing surface-to-volume ratio as the droplet size increases. With increasing droplet size due to hygroscopic growth, surface tension increases due to the dilution by condensing water. Countering this surface tension increase is the reduction owing to decreasing surface-to-volume ratio at larger droplet sizes, although this is insufficient to offset the concentration change from hygroscopic growth. Once enough water has condensed on the droplet, the surface tension approaches that of pure water (72 mN·m^−1^). With increasing initial surfactant concentration, an increasing fraction of droplet growth occurs with a surface tension <72 mN·m^−1^. The effects on critical radius (*R*_c_), critical supersaturation (*SS*_c_), and surface tension at activation (γ_c_), as predicted by the Köhler equation (2, 44), are shown in Fig. 5*C* and Table 1. When [Triton X-100]_tot,init_ = 1 mM, γ_c_ is equivalent to that for the reference case where [Triton X-100]_tot,init_ = 0 mM. Consequently, *R*_c_ and *SS*_c_ are not significantly affected by the presence of surfactant. Similarly, when [Triton X-100]_tot,init_ = 10 mM, γ_c_ = 64 mN·m^−1^, resulting in ∼10% changes to *R*_c_ and *SS*_c_. However, when [Triton X-100]_tot,init_ = 100 mM, droplet surface tension is predicted to be low during all of the droplet growth (γ_c_ = 55 mN·m^−1^), resulting in an *R*_c_ of 0.12 μm and an *SS*_c_ of 0.57%, representing a 50% change in *R*_c_ and a 37% change in *SS*_c_ relative to the scenario where [Triton X-100]_tot,init_ = 0 mM. When [Triton X-100]_tot,init_ = 1,000 mM, surface tension is at a minimum throughout growth, remaining 34 mN·m^−1^ at activation, thereby modifying *R*_c_ to 0.15 μm and *SS*_c_ to 0.25%. Such changes in *R*_c_ and *SS*_c_, particularly for the cases where [Triton X-100]_tot,init_ > 10 mM, could be climatically significant, as demonstrated by a simplistic estimate of the fractional change in marine warm cloud deck droplet number concentration (Δ*N*_d_/*N*_d,est_) and the resulting *IRE* (*IRE*_est_) due to the decreased *SS*_c_, both shown in Table 1 (*Methods*). For example, when [Triton X-100]_tot,init_ = 100 mM, *N*_d_ is predicted to increase by >30% and *IRE* to change by −1.6 W·m^−2^, similar in magnitude to the uncertainty in the aerosol indirect effect on radiative forcing. Although these values are estimates considering only ACI in a subset of cloud regimes in the Earth system (1), they are consistent with previous estimations of surfactant effects on *N*_d_ and *IRE* (4, 5). However, more realistic and accurate estimates of surface tension effects on climate require detailed sensitivity analysis in cloud resolving and properly parameterized global models across a more dynamic set of experimental conditions (6).

**Table 1.** Parameters for droplets with initial composition (at 0.05-μm radius) indicated in Fig. 5*A*

[Triton X-100]_tot,init_, mM	*R*_c_, µm	*SS*_c_, %	γ_c_, mN·m^−1^	Δ*N*_d_/*N*_d,est_	*IRE*_est_, W·m^−2^
0	0.076	0.91	67		
1	0.076	0.89	67	0.054	−0.22
10	0.085	0.82	64	0.088	−0.48
100	0.12	0.57	55	0.32	−1.6
1,000	0.15	0.25	34	0.99	−4.9

*R*_c_: critical radius, *SS*_c_: critical supersaturation, γ_c_: surface tension at activation, Δ*N*_d_/*N*_d,est_: estimated fractional change in *N*_d_, *IRE*_est_: estimated *IRE* when compared to the reference case.

Triton X-100 is likely to be representative of many atmospheric surfactants, as its CMC and γ_CMC_ are consistent with those reported in atmospheric aerosol (7, 20). Consequently, the monolayer partitioning model predictions suggest that the total surfactant concentration in particles must be at least on the order of several tens to 100 mM for there to be a substantial effect on cloud droplet activation. Gérard et al. (7, 29) recently reported total surfactant concentrations in collected ambient fine aerosol reaching several tens to hundreds millimolar across Europe. Moreover, surfactant concentrations are enhanced in smaller (<200-nm diameter) atmospheric particles (46). Such atmospheric observations combined with our results suggest surfactants in some cases will substantially impact the activation of atmospheric particles into cloud droplets. However, it may be unlikely for droplet surface tension to equal γ_CMC_ at *R*_c_, which emphasizes the importance of considering finite size-dependent surfactant partitioning to accurately estimate surface tension. These results indicate surfactant effects may be challenging to identify in ambient studies because surfactants may only significantly affect cloud droplet activation under specific scenarios (12). Consequently, these results underscore the need for further investigation of surfactant effects, exploring a broader range of model surfactants and cosolutes to develop a more refined understanding of surfactant effects on cloud droplet activation.

In conclusion, we provide direct measurements of the surface tensions of surfactant-coated, finite-sized picoliter droplets, which demonstrate that surface partitioning effects can result in droplet surface tensions up to 35 mN·m^−1^ larger (i.e., closer to the value for water) than the corresponding macroscopic solution owing to their high surface-to-volume ratio. Therefore, surface-bulk partitioning must be considered when reconciling finite-sized droplet surface tensions with macroscopic solution values. It is insufficient to simply use the macroscopic solution surface tension. We also demonstrate experimentally that surfactant partitioning in finite-sized droplets is substantially affected by the presence of a cosolute (glutaric acid, NaCl). Considering atmospheric aerosols are complex chemical mixtures containing inorganic salts and organic molecules, of which surfactants are an important component (7, 46), the surfactant–solute interactions and resulting size-dependent surface-bulk partitioning effects must be further explored.

The picoliter droplet surface tension measurements are rationalized in a monolayer partitioning model framework constrained only to well-established macroscopic solution measurements. The qualitative agreement between measurement and model strengthens our confidence in the application of surface tension models accounting for surface-bulk partitioning as well as highlights the necessity for further model development to predict more accurately the surface properties of complex aerosol particles. Improved predictive capabilities will enable better estimation of *N*_d_, ultimately reducing uncertainty in the indirect aerosol effect. The surface monolayer model predictions suggest surfactant concentrations on the order of several tens to 100 mM, consistent with reported atmospheric concentrations, are required to substantially affect *N*_d_ (i.e., modify *SS*_c_ by >20%). These predictions highlight the necessity of a more comprehensive understanding of the types and concentrations of surfactants in aerosol, as to date only a few studies in a few environments have been reported (7, 8, 10, 29, 46, 47). Global simulations of *N*_d_ have only rarely incorporated such surface partitioning models (5, 6), in part because partitioning effects had not been experimentally confirmed until this work. The broader implication of this work is that, despite the effects of surface-bulk partitioning, surfactants are still likely to impact cloud droplet activation in a range of atmospheric scenarios, with the effect varying greatly with local conditions including the aerosol size distribution, surfactant concentration, and cosolute identities. Detailed climate modeling studies (5, 6) are required to identify quantitatively the magnitude of these climate impacts.

## Methods

### Holographic Optical Tweezers.

The instrumental approach has been described in detail elsewhere and is only briefly summarized here (23, 39, 40). Aerosol droplets are produced from an aqueous solution containing known concentrations of solute and surfactant using a standard medical nebulizer (NE U22; Omron). Droplets of typically 5- to 10-μm radius are captured in individual optical traps. These two steerable optical traps were created by dynamically shaping the phase front of a continuous-wave 532-nm laser (Opus 3W; Laser Quantum) using a liquid crystal on silicon spatial light modulator (SLM) (X10468; Hamamatsu). The beam was expanded to fill the SLM display, which was conjugated to the back focal plane of a high-numerical-aperture microscope objective (Olympus ACH, 100×/1.25 oil) by a pair of condensing 4*f* telescopes. The trap separation was user controlled using a precalculated sequence of kinoforms (phase-only computer-generated holograms). A sufficiently small trap separation resulted in coalescence of the two trapped droplets into one composite droplet. Bright-field images to monitor droplet capture and coalescence were recorded with a camera (Dalsa Genie HM 640, complementary metal–oxide–semiconductor). Backscattered Raman light was imaged onto the entrance slit of a 0.5-m-focal-length spectrograph (Action Spectra Prop SP-2500; Princeton Instruments), dispersed by a 1,200 line pairs per millimeter grating onto a cooled charge-coupled device camera. The resulting Raman spectrum from a spherical droplet consists of a broad underlying Stokes band with superimposed resonant structure at wavelengths commensurate with whispering gallery modes, from which the radius, RI, and dispersion can be determined with accuracies better than 2 nm, 0.0005, and 3 × 10^−8^ cm, respectively (48). Elastically backscattered light arising from the coalescence event was measured using a silicon photodetector (DET 110; Thorlabs) and recorded using a low-load, high-bit-rate oscilloscope (HDO 6034-MS; LeCroy).

When the precursor droplets have sufficiently low viscosity, coalescence excites damped oscillations in droplet shape, the frequency of which (*ω*_*l*_) is described by Eq. **2** (41):

$$\omega_{l}^{2}=\frac{l\left(l-1\right)\left(l+2\right)\gamma }{a^{3}\rho },$$ [2]

where *γ* is the droplet surface tension, *l* is the oscillation mode order or characteristic deformation in droplet shape (here, *l* = 2), *ρ* is the droplet density, and *a* is the droplet radius. The frequency of these shape oscillations was ascertained by applying the fast Fourier transform to the time-dependent elastically backscattered light signal. The measured oscillation frequency (*ω*_l_*) was corrected for effects arising from droplet viscosity (49):

$$\omega_{l}^{*}=\sqrt{\omega_{l}^{2}-\tau_{l}^{-2}},$$ [3]

where *τ*_*l*_ is related to the dynamic viscosity (*η*), radius (*a*), and density (*ρ*) of the droplet by the expression (49)

$$\tau_{l}=\frac{a^{2}\rho }{\left(l-1\right)\left(2l+1\right)\eta }.$$ [4]

For the droplets analyzed in this study, the correction was generally very small (<2 mN·m^−1^). Note that this correction does not account for potential contributions from surface viscosity (50, 51), but we estimate such effects impact the retrieved surface tension by <10%.

Droplets containing one primary solute (either glutaric acid, 99%; Acros or NaCl, 99.9999%; Sigma) and one soluble surfactant (Triton X-100, electrophoresis grade; Fisher) were studied. Surfactant concentrations were sufficiently low that effects on the solution RI were minimal (<0.0005, measured with a refractometer). Therefore, the primary solute concentration (glutaric acid or NaCl) was measured directly via retrieval of the droplet RI through previously reported parameterizations for the primary solutes (23). To calculate the total surfactant concentration within a droplet, it was assumed that the known primary solute:surfactant ratio in the macroscopic solution was conserved after nebulization. This assumption is justified for the following three reasons. First, collection of nebulized aerosol (∼15 mL nebulized from a 30-mL solution) containing glutaric acid and Triton X-100 had the same RI and surface tension as the initial solution and the residual solution in the nebulizer, which is only expected if the glutaric acid:Triton X-100 ratio is conserved upon nebulization (*SI Appendix*, Table S1). Second, if the ratio were not conserved, one might expect substantial variability in retrieved surface tensions among measurements produced from solutions with the same primary solute:surfactant ratio. In fact, the surface tension values of droplets produced by nebulization of the same solution were uniformly consistent (<±3 mN·m^−1^) over several weeks even using different nebulizers, with clear and consistent differences in measured droplet surface tensions apparent only when the primary solute:surfactant ratio was changed. Third, in previous work with mixtures of glutaric acid and NaCl, the retrieved droplet RI and corresponding surface tension gave excellent agreement with model predictions and macroscopic solution measurements, indicating the relative ratio of glutaric acid and NaCl in solution was conserved upon nebulization (26, 52).

The slight discrepancy (<7 mN·m^−1^) between macroscopic solution and droplet surface tension measurements beyond the CMC may be due to the presence of the surfactant film on the surface of the droplet, which, as described above, can decrease the droplet oscillation frequency from its true value, resulting in a retrieved surface tension below the true value (50). Another possible explanation is that the composite droplet surface is slightly enriched in surfactant compared to a fully equilibrated surface. Coalescence produces a composite droplet with a smaller total surface area than the two initial droplets. If surfactant diffusion away from the composite droplet surface is slower than the timescale of the shape oscillation (10 to 100 μs), the composite droplet surface will not reestablish equilibrium, resulting in a tighter packing of surfactant molecules and a reduction in surface tension. In either case, the discrepancy would be most significant at values around the CMC. The magnitude of this discrepancy is always significantly less than the magnitude of the surface-bulk partitioning effects and would trend opposite to the surface-bulk partitioning effects.

### Macroscopic Solution Surface Tensiometry Measurements.

Surface tensions of all macroscopic solutions (which also served as the sources of the nebulized droplets) were measured using a Wilhelmy plate tensiometer (K100; Krüss).

### Macroscopic Solution Surface Tension Parameterisations.

Although Gibbs adsorption isotherm fits (provided in *SI Appendix*, Fig. S3 for comparison) quantitatively reproduce surface tension measurements for ternary systems, they are not adequate for implementation into the monolayer partitioning model, as the Gibbs adsorption isotherm cannot be extended to arbitrary bulk compositions needed for partitioning calculations. Therefore, a modified Szyszkowski–Langmuir parameterization was fitted to the macroscopic solution surface tension data for both the glutaric acid–Triton X-100 and the NaCl–Triton X-100 systems at 298 K (*SI Appendix*, Fig. S1). Ternary water–glutaric acid–Triton X-100 and water–NaCl–Triton X-100 systems (measured in this work; *SI Appendix*, Fig. S1), binary water–glutaric acid (previously measured by us) (23), and binary water–NaCl and water–Triton X-100 systems (from the literature) (53, 54) were used to constrain nonlinear least squares fits. Motivated by Fainerman et al. (55), various functional forms for the fits were tested. Eq. **5** was used to describe the glutaric acid–Triton X-100 data (SE of regression, SER = 0.545 mN·m^−1^; *SI Appendix*, Fig. S1*A*):

$$\gamma =\gamma_{\text{water}}-\frac{a_{1}\left[\text{Triton}\;\text{X}-100\right]+a_{2}\left[\text{glutaric acid}\right]+a_{3}\left[\text{Triton}\;\text{X}-100\right]\left[\text{glutaric acid}\right]}{\left[\text{Triton}\;\text{X}-100\right]+\left[\text{glutaric acid}\right]}\times \left\{\ln\left(1+b_{1}\left[\text{glutaric acid}\right]\right)+\ln\left(1+b_{2}\left[\text{Triton}\;\text{X}-100\right]\right)\right\}.$$ [5]

Eq. **6** was used to describe the NaCl–Triton X-100 data (SER = 1.07 mN·m^−1^; *SI Appendix*, Fig. S1*B*):

$$\gamma =\gamma_{\text{water}}-\frac{a_{1}\left[\text{Triton}\;\text{X}-100\right]+a_{2}\left[\text{NaCl}\right]+a_{3}\left[\text{Triton}\;\text{X}-100\right]\left[\text{NaCl}\right]}{\left[\text{Triton}\;\text{X}-100\right]+\left[\text{NaCl}\right]}\times \left\{b_{1}\left[\text{NaCl}\right]+\ln\left(1+b_{2}\left[\text{Triton}\;\text{X}-100\right]\right)\right\}.$$ [6]

The higher SER for NaCl–Triton X-100 arises due to poorer fits at high solute concentrations ([NaCl] > 2 M and [Triton X-100] > 3 mM), which are not important for the concentrations in the droplets studied here. Cross terms *a*_3_[surfactant][solvent] represent contributions from composition dependent molecular area per surfactant molecule due to surfactant–solvent interactions. In both cases, the underlying parameters (*a*_1_ and *b*_2_) for both fits to the macroscopic solution data (provided in *SI Appendix*, Table S2) agree with those given by Zdziennicka et al. (54) for the Szyszkowski–Langmuir equation within reported uncertainties for the limiting case of binary water–Triton X-100 solution (i.e., primary solute concentration = 0 M). At the limit of a water–glutaric acid solution (i.e., surfactant concentration = 0 mM), *a*_1_ and *b*_2_ agree with literature values (56). At the limit of a water–NaCl solution, the fit function predicts a linear dependence of surface tension on salt concentration with the resulting coefficient *a*_2_*b*_1_ slightly below literature values (57). The surface tension value used for pure water at 298 K was 72.0 mN·m^−1^.

Liquid-phase densities were estimated as those of ideal pseudobinary mixtures of aqueous glutaric acid/NaCl (58, 59) and Triton X-100, respectively, using a constant value of 1,070 kg·m^−3^ for the density of Triton X-100. The density and surface tension of pure, subcooled NaCl (required for Eq. **7**) were extrapolated from the correlations presented by Janz (60). These density and surface tension parameterisations served as the basis for the droplet surface tension model described below.

### Monolayer Partitioning Scheme for Finite-Sized Droplets.

The surface tension of aqueous droplets is calculated based on the droplet bulk composition, which is determined from the total composition by accounting for bulk-to-surface partitioning of surfactant molecules in the droplet. A more detailed description of the monolayer partitioning model is given in Malila and Prisle (43).

To determine the surface and bulk concentrations, the finite-sized droplet is conceptually divided into a surface and interior (bulk). The surface is considered as a finite-sized phase with a thickness *δ* = (6Σ_*i*_ *x*^*i*^_surface_ *v*_*i*_/π)^1/3^, corresponding to one layer of molecules, while the interior radius is *a* − *δ*. Partitioning of water, surfactant, and primary solute molecules between surface and bulk phases is calculated for a specified droplet radius *a* and surface thickness *δ*, assuming conservation of mass as well as liquid-phase densities corresponding to the respective surface and bulk phase compositions {*x*_bulk_} and {*x*_surface_}. The bulk composition is related to the surface tension by

$$\gamma \left(\left\{x_{\text{bulk}}\right\}\right)=\frac{\sum_{i}x_{\text{surface}}^{i}v_{i}\gamma_{i}}{\sum_{i}x_{\text{surface}}^{i}v_{i}},$$ [7]

where *x*^*i*^_surface_ are the surface mole fractions, *v*_*i*_ are the condensed phase molecular volumes, and γ_*i*_ are the pure compound surface tensions, respectively, for each component *i* in the droplet (here, water, glutaric acid or NaCl, and Triton X-100). The only experimental data used to constrain the model are the modified Szyszkowski–Langmuir parameterization of the macroscopic solution surface tension data (*SI Appendix*, Fig. S1) and the solution density.

The surface tension of pure, nonaqueous Triton X-100 is not known and therefore is approximated by γ_CMC_. Combined with Eq. **7**, this leads to the condition that, at the CMC, *x*_surface_ ≈ 1 for Triton X-100. In other words, the model assumes formation of a complete monolayer at the CMC. It is possible that the surface is not completely saturated when the CMC is approached in the bulk, in which case this assumption leads to a sudden jump in the modeled surface mole fraction *x*_surface_ of Triton X-100 (and also in surface thickness *δ*). Furthermore, because the volume and density of the surface and bulk phases depend nonlinearly on [Triton X-100]_tot_, the assumption of a pure monolayer can in some cases lead to numerical fluctuations in the model close to the CMC. However, the effect on model predictions is minor.

### Estimations of Cloud Droplet Number Concentration (*N*_d_) and *IRE*.

We estimate the effect of increased droplet surface tension from including surface-bulk partitioning in predictions of cloud droplet activation. The relationship between *N*_d_ and *SS*_c_ is calculated as

$$\frac{\Delta N_{\text{d}}}{N_{\text{d}}}=\frac{{\left(SS_{\text{C}}^{0}\right)}^{k}-{\left(SS_{\text{C}}\right)}^{k}}{{\left(SS_{\text{C}}\right)}^{k}},$$ [8]

where *SS*^0^_c_ and *SS*_c_ indicate the critical supersaturations determined for the baseline ([surfactant] = 0 mM, i.e., assuming surface tension close to that of pure water) and perturbed ([surfactant] > 0 mM) cases, respectively. For consistency, both are evaluated using the full monolayer partitioning model framework described above. Eq. **8** follows from the commonly assumed dependence *N*_d_ ≈ *cSS*^*k*^, where *c* is the number of CCN activated at 1% supersaturation and *k* ≈ 0.5 (4, 61).

Previous approaches have estimated Δ*N*_d_/*N*_d_ ≈ *k*Δ*SS*_c_/*SS*_c_ ≈ −(3*k*/2)Δγ_c_/γ_c_ from the Köhler equation for small changes in *N*_d_, while recognizing that a change in cloud microphysics due to a decrease in γ_c_ has the same effect as keeping γ fixed and increasing supersaturation *SS* (4). This simple approach has yielded results very similar to full climate model calculations (5) but becomes invalid when the presence of cosolutes and surfactant surface-bulk partitioning (as in this work) introduces a higher degree of nonlinearity between *SS*_c_ and γ_c_ (16, 18, 22). Therefore, we use Eq. **8**, which allows larger relative changes in *SS*_c_.

The change in cloud-top albedo (*A*) at constant liquid water content from including surface-bulk partitioning is then calculated from the change in cloud droplet number concentration (Δ*N*_d_) determined from Eq. **8** as (61)

$$\Delta A=A\left(1-A\right)\Delta N_{\text{d}}/\left(3N_{\text{d}}\right).$$ [9]

By using the common reference case of the change in cloud top albedo due to single reflection from the warm marine cloud deck (4, 61), we calculate the short-wave radiative effect as

$$IRE_{\text{est}}\approx -F_{0}f_{A}T_{a}^{2}\Delta A,$$ [10]

where *F*_0_ = 340 W·m^−2^ is the incoming solar flux at the top of the atmosphere. In Eq. **10**, *f*_*A*_ = 0.3 is the fractional coverage of marine stratus and stratocumulus clouds with *A* ≈ 0.5, and *T*_*a*_ = 0.76 is the transmittance of the atmosphere at visible wavelengths (61).

### Data Availability.

All data underlying the figures are provided through the University of Bristol Data Repository, data.bris (62).

## Supplementary Material

Supplementary File
