# Supplementary material for: The surface tension of surfactant-containing, finite volume droplets
Source: Proc Natl Acad Sci U S A. 2020 Apr 1;117(15):8335–43. doi: 10.1073/pnas.1915660117 (PMC7165431; doi:10.1073/pnas.1915660117)
Supplement: Supplementary File [file pnas.1915660117.sapp.pdf]

PNAS
www.pnas.org

Supplementary Information for

The Surface Tension of Surfactant-Containing, Finite Volume Droplets

Bryan R. Bzdek, Jonathan P. Reid, Jussi Malila, and Nønne L. Prisle

Bryan R. Bzdek, Jonathan P. Reid
Email: b.bzdek@bristol.ac.uk, j.p.reid@bristol.ac.uk

This PDF file includes:

Figures S1 to S3
Tables S1 and S2

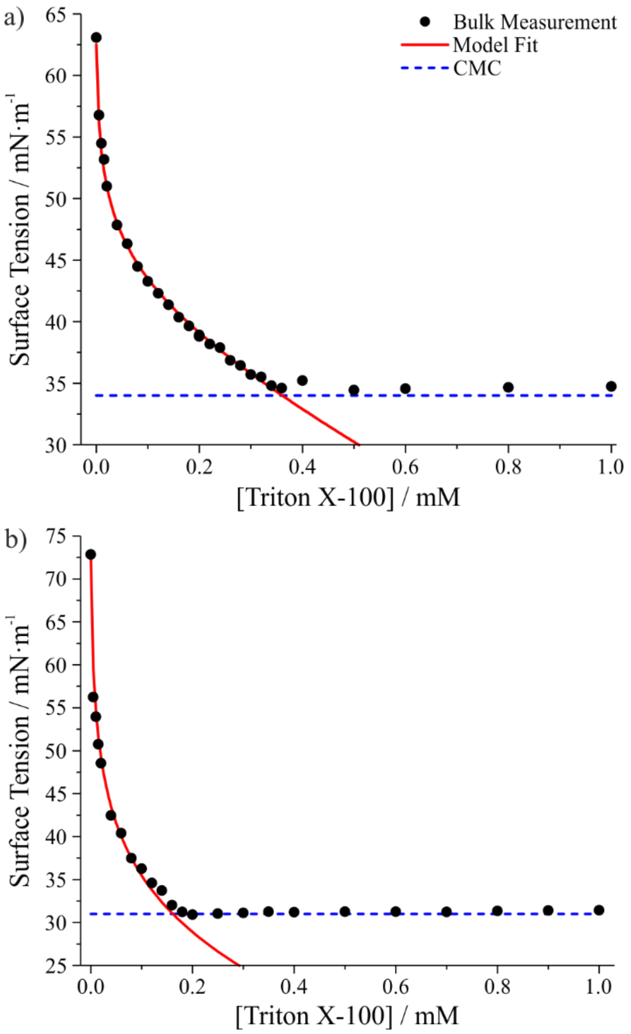

Figure S1: Macroscopic solution measurements of surface tension as a function of [Triton X-100] (= [Triton X-100]$_{tot}$ ≈ [Triton X-100]$_{bulk}$) a) for a solution of 0.9 M glutaric acid and b) for a solution of 0.5 M sodium chloride. The red curve is the modified Szyszkowski–Langmuir parameterisation of the experimental data fit (parameters in Table S1). The blue dashed line illustrates the surface tension beyond the CMC. Only the parameterisation of the experimental data below the CMC is used in the monolayer partitioning model for finite-sized droplets.

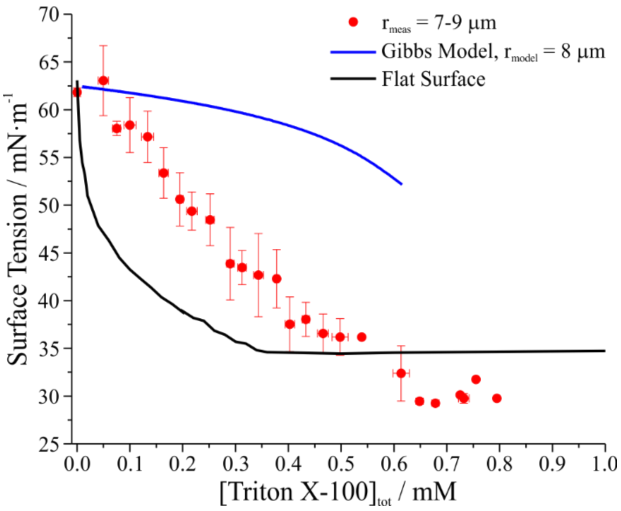

Figure S2: Comparison of picolitre droplet (~7-9 μm radius) surface tensions to macroscopic solution surface tensiometry measurements and a Gibbs partitioning model (1) as a function of [Triton X-100]$_{tot}$ for droplets nebulised from an aqueous solution containing 0.9 M glutaric acid. Droplet measurements are averaged to 0.03 mM bins. Uncertainty bars represent the standard deviation of the mean. As discussed in the main text, the Gibbs partitioning model overpredicts surfactant partitioning, resulting in predicted droplet surface tensions much higher than experimentally measured.

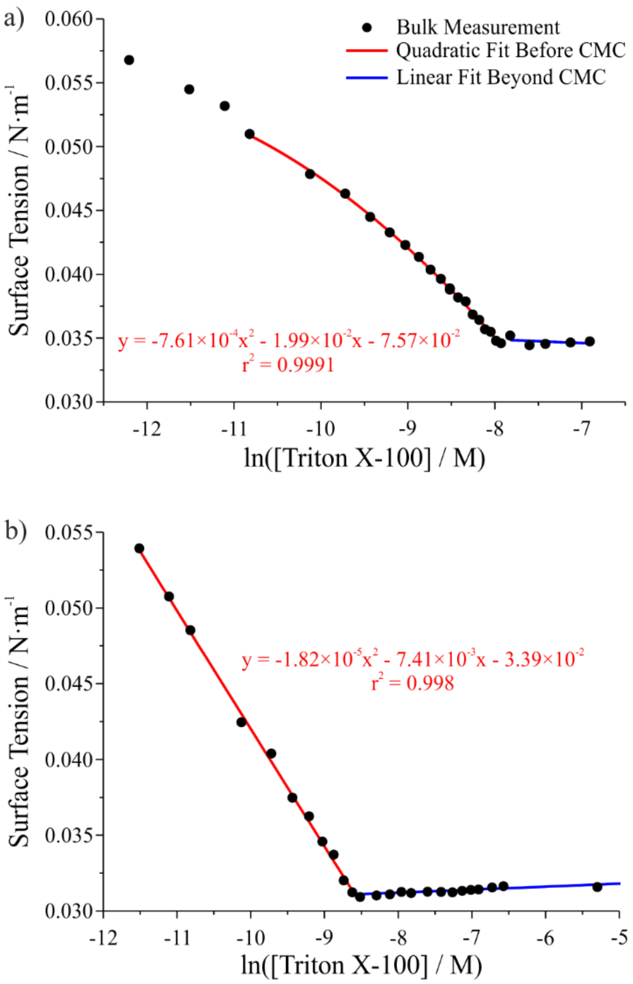

Figure S3: Concentration dependent macroscopic solution surface tension data for a) glutaric acid-Triton X-100 and b) NaCl-Triton X-100 with Gibbs adsorption isotherm fits for comparison to the modified Szyszkowski-Langmuir parameterisations (see Figure S1).

Table S1: Nebulisation of an aqueous solution of 0.9 M glutaric acid and 0.1 mM Triton X-100 gives the same surface tension and RI as the original solution.

Sample	Refractive Index	Surface Tension / mN·m^{-1}
Original Solution	1.3464	45.28 ± 0.08
Nebulized Fraction	1.3466	45.41 ± 0.07
Residual Fraction Not Nebulised	1.3464	45.74 ± 0.06

Table S2: **Parameters used in the surface tension fitting and standard errors of regression (SER) for the fits.**

Parameter	Glutaric acid-Triton X-100	NaCl-Triton X-100
a_1 / N·m^{-1}	7.09×10^{-3}	7.16×10^{-3}
a_2 / N·m^{-1}	4.16×10^{-3}	8.56×10^{-3}
a_3 / N·M^{-1}·m^{-1}	1.92866	1.26059
b_1 / M^{-1}	9.7364	-1.54×10^{-1}
b_2 / M^{-1}	7.02×10^{5}	7.15×10^{5}
SER / N·m^{-1}	0.545×10^{-3}	1.07×10^{-3}

Reference:

1. Prisle NL, Raatikainen T, Laaksonen A, Bilde M (2010) Surfactants in cloud droplet activation: Mixed organic-inorganic particles. *Atmos Chem Phys* 10(12):5663–5683.
